# Strategies for management of stress and burnout among healthcare professionals in Brazil

**DOI:** 10.47626/1679-4435-2022-653

**Published:** 2023-02-03

**Authors:** Marcos Adriel Balbinot, Maiara Bordignon

**Affiliations:** 1 Curso de Graduação em Enfermagem, Universidade do Contestado, Concórdia, SC, Brazil; 2 Universidade Federal do Paraná, Toledo, PR, Brazil

**Keywords:** occupational stress, burnout, psychological, health personnel, occupational health., estresse ocupacional, burnout, profissionais da saúde, saúde do trabalhador.

## Abstract

The objective of this study was to map evidence in the scientific literature regarding the implementation and impact of strategies for prevention and management of stress and/or burnout among healthcare professionals in Brazil. This is a Scoping Review carried out using search terms and Boolean operators to search the databases Latin American and Caribbean Health Sciences Literature (via Virtual Health Library), Scientific Electronic Library Online, and Medical Literature Analysis and Retrieval System Online (via PubMed). The publication period was from 2010 until the dates on which searches were run. A manual search and searches of the reference lists of selected publications were also conducted. Initially, 317 studies were identified and 14 studies were included in the final sample. The studies highlight the implementation of strategies for prevention and management of stress and/or burnout among healthcare professionals in Brazil together with their results. There was evidence of use of integrative and complementary practices, with emphasis on auriculotherapy, as well as stress reduction programs, and care-educational strategies. This review brings together possibilities for prevention and management of stress and burnout, presenting strategies and their results in the target population.

## Abstract (pt)

O objetivo do estudo foi mapear na literatura científica evidências sobre a implementação de estratégias para prevenção e manejo de estresse e/ou burnout e seu impacto entre profissionais de atenção à saúde no Brasil. Trata-se de uma revisão de escopo realizada nas bases de dados Literatura Latino-Americana e do Caribe em Ciências da Saúde via Biblioteca Virtual em Saúde, Scientific Electronic Library Online e Medical Literature Analysis and Retrievel System Online via PubMed a partir da aplicação de termos e operadores booleanos. O período de publicação foi considerado de 2010 até a aplicação das estratégias de busca. Também foram realizadas buscas manual e na lista de referências das publicações selecionadas. Inicialmente, foram identificados 317 trabalhos, mas apenas 14 estudos compuseram a amostra final. Os estudos destacam a implementação de estratégias para a prevenção e o manejo de estresse e/ou *burnout* entre profissionais de atenção à saúde no Brasil juntamente com os seus resultados. Evidenciou-se o uso de práticas integrativas e complementares, com destaque para a auriculoterapia, bem como de programas de redução do estresse e estratégias cuidativo-educacionais. Esta revisão reúne possibilidades para a prevenção e o manejo de estresse e *burnout* ao apresentar estratégias e seus resultados junto à população-alvo.

## INTRODUCTION

Nowadays, stress has a considerable impact on people’s health status and on quality of life.^^1^^ Within this context, the issue of occupational stress merits special attention and has been the object of many studies in a wide range of occupational areas or professional activities.^^2^-^4^^ There is evidence in the literature that occupational stress has a negative impact on health-related quality of life.^^5^^ It is also known that chronic exposure to interpersonal/emotional stressors at work can lead to burnout syndrome.^^6^-^7^^

It should be noted that burnout syndrome is a combination of three dimensions, specifically: (i) emotional exhaustion, (ii) depersonalization, and (iii) ineffectiveness (lack of personal accomplishment).^^6^,^7^^ Exhaustion is expressed as feelings that represent the excessive effort a worker has to make to perform their activities, resulting in exhaustion of emotional and physical resources.^^7^^ Depersonalization encompasses insensitive or excessively detached behavior at work, while the lack of personal accomplishment dimension constitutes a person’s self-assessment with relation to their competencies, achievements, and work productivity.^^7^^ These dimensions are associated with a set of signs or symptoms the demonstrate the worker’s burnout, including lack of energy, tiredness, irritability, behavior considered inadequate with relation to clients, and reduced productivity and personal achievement.^^6^,^7^^

The occurrence of burnout has most frequently been associated with professions linked to serving and caring for people, such as education and medical care.^^6^^ In these areas, professionals perform daily activities that require continuous and intense personal and emotional contact and, depending on the characteristics of their work, for example workload, professional autonomy, remuneration, and interpersonal relationships, they may be more or less vulnerable to burnout.^^6^^

Data demonstrate that stress and burnout are both phenomena present among health professionals.^^8^-^11^^ In a study conducted with nursing professionals at a University Hospital in Minas Gerais, Brazil, 71.8% of professionals were exposed to occupational stress.^^9^^ Another study with Brazilian intensive care specialist physicians found a 61.7% prevalence of burnout, considering a high level of at least one of its dimensions, and in 5% of the sample all three dimensions were at high levels simultaneously.^^11^^ In addition to its prevalence, the literature also reveals the negative impact of burnout on workers, on their professions and employers, and on society.^^1^,^5^-^7^,^12^-^14^^

Given the nature of the activities performed by health professionals and their importance to society, it is clear that these workers need to be in good emotional health, be competent at their roles, and feel personal accomplishment, in order that they can provide good quality care. Along the same lines, bearing in mind the effects of stress and of burnout, both on health professionals and on the populations they care for,^^1^,^5^-^7^,^12^-^14^^ it is also clear that strategies or actions aimed to prevent and manage these phenomena are extremely important.

This being so, the objective of the present study was to map evidence in the scientific literature related to the implementation and impacts of strategies for prevention and management of stress and/or burnout among healthcare professionals in Brazil.

## METHODS

A Scoping Review Study following the structure described by Arksey & O’Malley,^^15^^ including five phases: (i) identify the research question; (ii) identify relevant studies, (iii) select studies; (iv) map the data; and (v) synthesize and report the results.

As a starting point, the first step was to define the following research question: Is there evidence related to the implementation and impact of strategies for prevention and management of stress and/or burnout among healthcare professionals in Brazil?

This research question was used to construct search strategies to identify relevant studies (the second step in the review).^^15^^ The following keywords were chosen for the search: Estresse Ocupacional, Burnout, Brasil, Occupational Stress, Brazil, and Brazilian, on the basis that using these keywords would return publications related to the research question. Searches for studies were run on the databases LILACS (Literatura Latino-americana e do Caribe em Ciências da Saúde) via the Biblioteca Virtual em Saúde (BVS), SciELO (Scientific Electronic Library Online), and MEDLINE (Medical Literature Analysis and Retrieval System Online), via PubMed, using these keywords and Boolean operators (see Table 1).

**Table 1:** Description of resources utilized to search for publications on the databases

Database	Strategies^*^	Filters
LILACS (BVS)	(tw:(Estresse Ocupacional)) OR (tw:(Burnout)) AND (tw:(Brazil))	Language: Portuguese, English, and Spanish 2010 to 2020
SciELO	(ab:(estresse ocupacional)) OR (ab:(burnout)) AND (ab:(Brazil))	Language: English, Spanish, and Portuguese From 2010 (up to 2019, 2020 was not yet available)
MEDLINE (PubMed)	(((Occupational Stress[Title/Abstract]) OR Burnout[Title/Abstract])) AND ((Brazil[Title/Abstract]) OR Brazilian[Title/Abstract])	Language: English, Portuguese, and Spanish Humans 01/01/2010 to 27/03/2020

* Searches conducted on March 27, 2020.

BVS = Virtual Health Library; LILACS = Latin American and Caribbean Health Sciences Literature; MEDLINE = Medical Literature Analysis and Retrieval System Online; SciELO = Scientific Electronic Library Online.

Additionally, manual searches were run using the Google search engine, in order to identify additional studies that fell within the scope of the review. Moreover, the references of the publications selected were analyzed, adding four more publications to the final sample. These different strategies were utilized with the objective of contributing to identification of relevant studies.^^15^^

In the next step, study selection,^^15^^ the titles and abstracts of all of the studies returned in the searches were analyzed by two reviewers. The following inclusion and exclusion criteria were applied to select the studies:

Inclusion criteria: i - research articles, reports of experiments or dissertations reporting on the implementation of strategies and evaluation of their impact for prevention and management of stress and/or burnout among healthcare professionals in Brazil; ii - published from 2010 up to the search date - March 27, 2020 (Table 1); iii - published in English, Portuguese, or Spanish.

Exclusion criteria: i - literature reviews, books, book chapters, publications in summary form or others not referenced in the inclusion criteria, and studies for which it was not possible to clearly identify the context in which the research was conducted from the published text; and ii -publications that were repeated or not fully identified.

**Figure 1:**
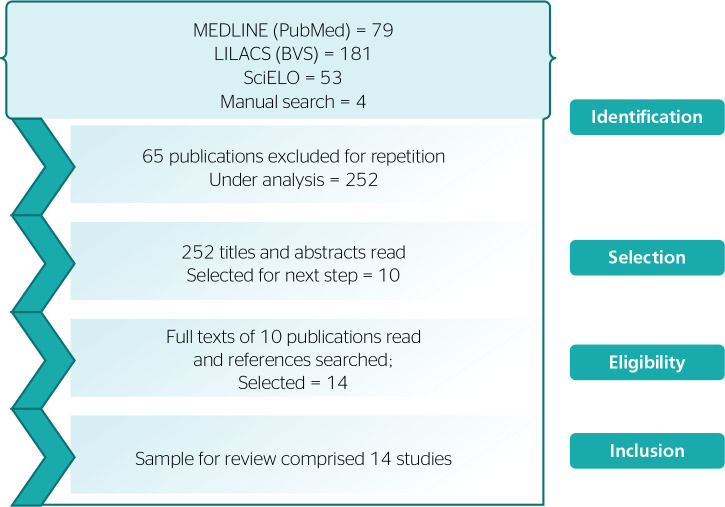
Process of selection and inclusion of studies following the recommendations of the Preferred Reporting Items for Systematic Reviews and Meta-Analyses (PRISMA).^^16^^

Figure 1 illustrates the process on which this review was based.

BVS = Virtual Health Library; LILACS = Latin American and Caribbean Health Sciences Literature; MEDLINE = Medical Literature Analysis and Retrieval System Online; SciELO = Scientific Electronic Library Online.

The data from the 14 studies were mapped after extraction of characteristics such as: authors, year and journal of publication, objectives, type of study and strategy reported, study participants and setting, instruments/assessments, and summary of results.

## RESULTS

It was found that the publications selected were published from 2012 to 2019 and all were in article format. The studies were predominantly conducted in hospital settings (78.6% of the studies). Other settings included Primary Care and the Fire Service. The majority of articles were published in Brazilian periodicals, and 21.4% were published in foreign periodicals (Table 2).

**Table 2:** Characteristics of the studies reviewed

Study details	Objectives	Type of study and strategy reported	Participants and setting	Instruments/assessments	Summary of results
Jacques et al.^^30^^ Revista Brasileira de Enfermagem	To compare occupational stress levels between nursing workers from the surgical center before and after a “wellbeing room” intervention	Quasi-experimental study. Strategy: “Wellbeing room” program. Duration: 6 months, in a room at the workplace. Activities included: occupational gymnastics, “beauty day”, auricular acupuncture, and lectures and workshops focusing on management of stress and coping with it	60 nursing workers, surgical center, teaching hospital in South Brazil	Questionnaire on sociodemographic and occupational characteristics; Demand-Control-Support Questionnaire Pre and post-intervention, single group	There was reduced demand, increased control and increased social support received at work in all occupational nursing classes, but differences did not attain statistical significance. Occupational stress levels reduced, but the reduction was not statistically significant.
Santos et al.^^24^^ Explore	To evaluate the effects of a Stress Reduction Program, including mindfulness and loving kindness meditation with nursing professionals working at a Brazilian hospital	Pilot study, mixed model (quantitative and qualitative methods). Strategy: Stress Reduction Program. Duration: 6 weeks. Educational intervention comprising 24 1-hour group sessions, including attention and concentration training, body examination, anchor breathing, informal mindfulness, and loving kindness meditation, plus discussions.	13 nursing workers at a University Hospital in São Paulo	Perceived Stress Scale, Maslach Burnout Inventory, Beck Depression Inventory, Trait-State Anxiety Inventory, Life Satisfaction Scale, Self-Compassion Scale, World Health Organization Quality of Life instrument-Abbreviated version; Stress at Work Scale. Interview in groups. Pre and post-intervention and follow-up	Significant reductions were identified between pre and post-intervention scores for perceived stress, burnout, depression, and anxiety, but there were no significant differences between post-intervention and follow-up scores. The quality of life assessment showed significant increase in the physical and psychological domains in post-intervention scores, which were maintained during follow-up. The qualitative results indicated improvements in reactivity to interior experience, more attentive perception of internal and external experiences, better attention to and awareness of actions and attitudes at every moment, and a positive influence of the program on nursing activities.
Leão et al.^^17^^ Plos One	To evaluate the impact of a self-care intervention mediated by the senses on levels of stress, self-esteem, and wellbeing among health professionals in a hospital setting	Randomized, controlled, open, clinical trial. Strategy: Self-care intervention mediated by the senses. Included three intervention groups with daily body moisturizing (except the face) using odorless cream (single-sense) or perfumed cream (dual-sense), the second of which was combined with audiovisual material before and during the procedure (multi-sense), and a control group. Duration: 30 days	93 female healthcare or health-related workers, at a private philanthropic hospital in the city of São Paulo	Sociodemographic, occupational, and self-care questionnaire; Lipp Stress Symptoms Inventory for Adults; Rosenberg Self-esteem Scale; Life Satisfaction Scale; Positive and Negative Affect Scale, plus samples of salivary cortisol and examination by a dermatologist at start and end of intervention. Start of study, after 15 days, after 30 days (final) and at 30-day follow-up	The dual-sense intervention group exhibited lowest stress, in addition to increased life satisfaction and positive affect and reduced negative affect. The multi-sense intervention group exhibited improved self-esteem and reduced cortisol levels after 30 days of the a intervention, and the control group only exhibited changes in cortisol levels.
Kurebayashi et al.^^18^^ Revista da Escola de Enfermagem da USP	To evaluate the stress levels of the nursing team at a hospital and analyze the effectiveness of auriculotherapy with needles and seeds	Randomized, controlled, clinical trial. Strategy: Auriculotherapy with needles or seeds Duration: eight sessions, from 5 to 10 minutes per session, one per week, targeting the Shenmen, kidney, and brain stem points. With a control group.	75 professionals from the nursing team at the USP University Hospital	Stress Symptoms List; sociodemographic questionnaire Baseline, before treatment, after 4 sessions, after 8 sessions, 15 days after completion.	Auriculotherapy treatment with needles and seeds reduced stress levels, with better results for needles than for seeds, and for individuals who had a high stress score. After the end of the intervention, the positive effect was maintained for 15 days.
Santiago et al.^^25^^ Complemen-tary Therapies in Clinical Practice	Conduct a feasibility assessment of a Stress Reduction Program based on mindfulness for primary care workers in the Brazilian unified health system.	Pilot mindfulness program. Before and after design without control group. Strategy: Stress Reduction Program based on mindfulness. Duration: Four weekly meetings, during November. 1 hour and 30 minutes each	26 primary care workers, municipal district of Biguaçu	Direct observations and four self-report questionnaires were developed, including presentations for specialists. Indicators, subdimensions and dimensions, assessment matrix, and judgment matrix	Implementation of a program was judged to be feasible. There is high demand for stress reduction interventions in this setting, and participant and management acceptance was excellent. Two main barriers were the need for a shortened version of the program and the low retention rates.
Kurebayashi et al.^^19^^ Revista Latino-Americana de Enfermagem	To assess auriculotherapy, for reduction of stress levels, and analyze the principle domains of coping that are changed after treatment	Randomized clinical trial. Strategy: three groups (control, needles, seeds). Duration: Eight sessions, 1 per week, 5 to 10 minutes each, targeting the Shenmen, kidney, and brain stem points. No intervention in the control group	75 nursing professionals at a teaching hospital in São Paulo	Sociodemographic data questionnaire Vasconcelos Stress Symptoms List; Folkman and Lazarus Coping Strategies Inventory. At baseline, before treatment, after 4 and 8 sessions, and 15 days after completion	Auriculotherapy treatment was positive for reduction of stress levels, with better results with semi-permanent needles than with seeds. Use of the domains withdrawal, confrontation, and social support reduced in the intervention groups after treatment, suggesting that auriculotherapy, both with needles and with seeds, can have positive impacts on improving coping strategies in the nursing team.
Melo & Carlotto^^26^^ Estudos de Psicologia	To present a proposal for a preventative program for management of stress and burnout Syndrome among professional firefighters	Report of the experience of an intervention. Strategy: Preventative program for management of stress and burnout Syndrome. Duration: Five fortnightly, 2-hour sessions for 3 months. The proposal included themes, objectives, and techniques	5 firefighters from the 1st Command of the State of Rio Grande do Sul	Sociodemographic and occupational data collection, observation, and field diary, Impact of Training on Work Assessment questionnaires and evaluation of the intervention at completion	It was concluded that the program enabled participants to better deal with occupational stressors.
Moreira et al.^^27^^ Revista de Enfermagem do Centro Oeste Mineiro	Describe educational workshops for reduction of factors that generate professional burnout in a family health nursing team in Belo Horizonte	Report of experience, descriptive study. Strategy: Implementation of three educational workshops with theoretical-practical activities Duration: mean duration of one and a half hours	Health professionals from a Family Health Center in Belo Horizonte, Minas Gerais	A scale on an individual form for participants to evaluate. Final evaluation by participants	The participants evaluated the workshops as relevant, since they provided opportunities in a playful and participatory manner for sharing experiences, interactions, reflections, and, primarily, for strengthening the team.
Taets et al.^^28^^ Revista Brasileira de Enfermagem	To determine the effects of a music therapy program on the stress levels of health professionals	Therapeutic intervention. Strategy: music therapy sessions. Music therapy techniques: improvisation and musical re-creation	34 volunteers from different professional areas at a private hospital in the city of Rio de Janeiro	Lipp Stress Symptoms Inventory for Adults; Semistructured Questionnaire for assessment of stress. Pre and post-intervention, single group	Researchers highlighted the statistically significant reduction in stress level after the music therapy program, concluding that the music therapy program was effective for reducing stress levels.
Ravaglio et al.^^29^^ Revista Brasileira de Terapias e Saúde	To assess the influence of auriculotherapy on stress levels among nursing professionals working in a pediatric ICU	Prospective study with a quantitative approach. Strategy: professionals who exhibited stress underwent up to six sessions of auriculotherapy with fifteen day intervals between sessions	46 professionals, 35 with a certain level of stress and 28 who underwent auriculotherapy. Nursing professionals working in a pediatric ICU at a children’s hospital in South Brazil	Lipp Signs and Symptoms of Stress Inventory and questionnaire on demographic information and perceptions of the technique Pre and post-intervention	67.9% of the professionals who underwent auriculotherapy improved. The authors concluded that auriculotherapy proved to be a technique that could help to reduce signs and symptoms of stress in this population.
Kurebayashi & Silva^^21^^ Revista Brasileira de Enfermagem	To assess efficacy of auriculotherapy to improve quality of life and reduce stress in a nursing team	Randomized, controlled, clinical trial. Strategy: three groups - control, protocol (Shenmen, brain stem, kidney, and liver Yang 1 and 2 points) and no protocol (same number of sessions and points, but chosen in accordance to response to treatment). Duration: Twelve sessions (twice per week), 5 to 10 minutes each, semi-permanent needles	175 nursing professionals at a general hospital in São Paulo	Vasconcelos’ SSL. SSL; questionnaire with sociodemographic data, TCM diagnostic form, and the SF36v2. Baseline, after 12 sessions, and follow-up (30 days)	The individualized auriculotherapy (the no protocol group) had superior effects to auriculotherapy with a protocol for reduction of stress and improvement of quality of life. In comparison with the fixed protocol, the individualized treatment was able to extend the reach of the Chinese auriculotherapy technique to reduce stress levels and improve quality of life in nursing professionals.
Kurebayashi et al.^^22^^ Revista Eletrônica de Enfermagem	Identify TCM diagnoses of stress symptoms that responded to treatment with auriculotherapy	Randomized clinical trial. Strategy: 3 groups (control without treatment, auriculotherapy with needles, and auriculotherapy with seeds). Duration: Eight sessions (one per week), targeting Kidney, Shenmen, and brain stem points	75 professionals from the nursing team at a Teaching hospital in São Paulo	Vasconcelos’ SSL. SSL1 or Baseline, SSL2 (at fourth session), SSL3 (eighth session) and SSL4 (15-day follow-up )	Auriculotherapy reduced stress and needles had better results than seeds. Twenty-one symptoms of stress exhibited significant differences from baseline to after eight sessions in the needle group, but the main symptoms were related to tachycardia, abnormal blood pressure, rage, insecurity, and nightmares. Eight symptoms changed from baseline to after treatment with seeds, the most important being: muscles always tense, feeling like fainting, tearful eyes, and blurred vision.
Kurebayashi et al.^^20^^ Acta Paulista de Enfermagem	To assess efficacy of auriculotherapy with semi-permanent needles on the stress levels of a nursing team	Single-blind, controlled, randomized clinical trial. Strategy: three groups: group 1 (control - no intervention), group 2 (less experienced therapists) and group 3 (more experienced therapists). Duration: Eight sessions of auriculotherapy with semi-permanent needles (1 per week), from 5 to 10 minutes, targeting the Shenmen, kidney, and Brain stem points	49 nursing professionals from a University Hospital in São Paulo	Covolan’s SSL; sociodemographic data questionnaire. Before treatment, after four sessions, after eight sessions and 15 days after completion (follow-up)	Auriculotherapy with more experienced therapists effectively reduced stress in nursing professionals.
Kurebayashi & Silva^^23^^ Revista Latino-Americana de Enfermagem	To assess the efficacy of auriculotherapy, with and without a protocol, for reduction of stress levels in a e nursing team	Single-blind, controlled, randomized clinical trial. Strategy: control group (without intervention), group with a protocol and group without a protocol. Duration: Twelve sessions (twice per week), from 5 to 10 minutes each, targeting the Shenmen, brain stem, kidney, and liver Yang 1 and 2 points	75 nursing professionals from a private hospital in São Paulo	Vasconcellos’ SSL; questionnaire on sociodemographic data and presence or absence of prior comorbidities. Baseline, after 12 sessions, and 30 days after completion (follow-up)	Auriculotherapy with and without a protocol were both effective for reduction of stress levels. The group without a protocol had better results than the group with a protocol.

SSL = Stress Symptoms List; TCM = Traditional Chinese Medicine; USP = Universidade de São Paulo; ICU = intensive care unit.

The most common type of study was clinical trial, of which there were seven, all conducted in hospitals, six with nursing professionals and one with health professionals and workers from related areas.^^17^-^23^^ There were two pilot studies focused on nursing professionals and primary care workers;^^24^,^25^^ and there were two reports of experience with interventions, one with a group of firefighters and another with primary care workers.^^26^,^27^^ One study administered a therapeutic intervention to volunteers at a private hospital;^^28^^ another study assessed the influence of auriculotherapy on stress among nursing professionals working in intensive care units;^^29^^ and one study employed a quasi experimental model with nursing professionals at a University Hospital^^30^^ (Table 2).

With regard to the strategies employed, actions were implemented to reduce stress using a range of different practices, such as organizing a wellbeing room at the workplace,^^30^^ educational workshops targeting prevention and management of stress, burnout, and professional exhaustion,^^26^,^27^^ and stress reduction programs including mindfulness practices and loving kindness meditation.^^24^,^25^^ Other strategies used were auriculotherapy with seeds and needles,^^18^-^23^,^29^^ music therapy for stress reduction,^^28^^ and a self-care intervention mediated by the senses^^17^^ (Table 2).

Authors reported that these interventions yielded positive results in terms of stress reduction or were rated positively by participants, although not all studies observed statistically significant stress reduction.^^17^-^30^^ It was observed that these interventions could also influence other dimensions; for example, increasing quality of life and life satisfaction, and strengthening teams.^^17^,^21^,^24^,^27^^

Table 2 lists the characteristics of the studies reviewed, including their objectives, methodological or procedural aspects, and summaries of their results.

## DISCUSSION

The Brazilian studies included in the final sample of this review reported different strategies for prevention/management of stress and burnout among healthcare professionals. Of the strategies implemented, use of integrative and complementary practices stands out. These types of treatments have been gaining space in Brazil, particularly since 2006, when the National Policy on Integrative and Complementary Practices was approved within the scope of the country’s Unified Health System (SUS - Sistema Único de Saúde).^^31^^

In this context, a study with hospital nursing professionals demonstrated that auriculotherapy contributed to reduction of stress levels and/or was capable of minimizing its signs and symptoms.^^18^-^23^,^29^^ In another study, with a different protocol to previous studies, auricular acupuncture was used in a stress reduction program together with other strategies, but in this case the reduction in stress was not statistically significant, although the nursing team’s occupational stress levels were lowered.^^30^^

Music therapy is another integrative and complementary practice that is now covered by the SUS^^32^^ and researchers report that the practice has benefits for stress reduction.^^28^^ Authors claim that music therapy is not only effective for stress, but also for coping with situations that generate feelings such as anguish and anxiety, contributing to people’s wellbeing and to patient-professional interaction.^^33^,^34^^ As such, it is clear that integrative and complementary practices are being used both for stress reduction and to reduce anxiety, pain, and depressive symptoms.^^35^^ Use of these practices is justified, among other reasons, by the possibility of spiritual wellbeing, improved mood, increased compassion, and improved sleep.^^35^^

Another practice mentioned in these studies was mindfulness,^^24^,^25^^ which, in addition to its implications for stress reduction,^^24^,^25^^ has also been described as having positive effects on vitality, emotional regulation, and life satisfaction.^^36^^

This review also identified implementation of educational strategies aimed to aid in understanding stress and burnout and contribute to coping with these situations or conditions.^^26^,^27^,^30^^ The strategies employed included educational workshops, lectures, or preventative programs with activities of an educational nature.^^26^,^27^,^30^^

It is therefore appropriate to stress that discussions related to care-educational technologies are recent, since for a long time they were dealt with separately, i.e. either care technologies or educational technologies.^^37^^ Nowadays, care-educational technologies are considered an innovative option for design of products and processes, since educating and caring do not have to be dissociated.^^37^^

It should be emphasized that these studies demonstrated many consequences of stress and burnout, such as physical and psychological symptoms among workers, impairment of quality of life, and negative impacts on work processes and on care.^^1^,^13^,^14^^ Working from these considerations and the fact that this subject and its implications have only been discussed recently, emphasis is put on the importance of focusing on strategies that demonstrate efficacy and feasibility for reduction of stress and burnout among healthcare professionals.

It is also important to recognize the relevance of new studies describing research conducted and experiences with caring for the health of workers and students on healthcare courses in the realm of prevention and promotion, in addition to provision of care and rehabilitation when conditions have already set in.^^38^^ This is because studies undertaken with nursing and medicine students have demonstrated that many students in these areas already have elevated stress levels and conditions suggestive of burnout before they finish their academic training.^^39^,^40^^ It is understood that if these individuals are already suffering burnout while still in academic training, it is possible that in the future they will be less empathetic and less attentive to the needs of the users of health services, which can trigger reduced quality of care and sickness among the professionals themselves.^^40^^

It is valid to point out that this review has its limitations. Although attempts were made to ensure identification of relevant studies using several different means, as recommended by the method adopted,^^15^^ the strategies employed may not have been sufficient to identify other studies that make contributions on the subject, but were not included. Moreover, evaluation of the quality of evidence is not one of the objectives of the Scoping Review method.^^15^^

## FINAL COMMENTS

This Scoping Review collected recent Brazilian studies that report on strategies for prevention and management of stress and/or burnout among healthcare professionals and highlighted their results using both objective and subjective approaches. The review thus presents possibilities for prevention and management of stress and burnout, describing strategies and their results in healthcare professionals in Brazil. Analysis of prior experiences may offer a guide for future studies or innovative actions.

It was found that published reports exist of research, interventions, and experiences, that describe strategies designed to prevent and intervene in stress and burnout. Among the results reported, use of integrative and complementary practices, educational strategies, and programs for stress reduction stand out. It is considered that, taken together, these publications offer a wider view of strategies that have been implemented and assessed, facilitating analysis of their effects and feasibility. Strategies for prevention and management of stress and burnout are especially necessary, bearing in mind the implications of both these phenomena and their current prevalence, particularly in healthcare settings.
